# The Dispensary Keys

**Published:** 1912-03-23

**Authors:** W. B. Fitch

**Affiliations:** Dispenser. St. Bartholomew's Hospital, Rochester.


*To the Editor of* The Hospital.

### THE DISPENSARY KEYS.

*To the Editor of* The Hospital.

Sir,—Would you permit me to ask if it is customary or desirable that the matron of a hospital should have the keys of the dispensary?

I have protested strongly without avail, and the resident medical officers have supported me, as the following letter, signed by the house surgeon and the house physician clearly shows :—

"You may certainly count on any support we can give in this matter. We consider it absurd that any unqualified person, even the matron, should have access to drugs in the dispensary. There is no difficulty in obtaining the necessary drugs from us, and we think this is the proper way in which affairs of this kind should be managed."

We claim to be the oldest hospital in the world, being founded by Gundulph, Bishop of Rochester (1078), and it appears to be rather late to make this innovation.—I remain, Sir, yours faithfully,

W. B. Fitch, Dispenser.

St. Bartholomew's Hospital, Rochester.

